# Propagation of Pacemaker Activity and Peristaltic Contractions in the Mouse Renal Pelvis Rely on Ca^2+^-activated Cl^−^ Channels and T-Type Ca^2+^ Channels

**DOI:** 10.1093/function/zqac041

**Published:** 2022-08-24

**Authors:** Nathan Grainger, Cameron C Shonnard, Sage K Quiggle, Emily B Fox, Hannah Presley, Robbie Daugherty, Matthew C Shonnard, Bernard T Drumm, Kenton M Sanders

**Affiliations:** Department of Physiology and Cell Biology, University of Nevada, Reno School of Medicine, 1664 North Virginia Street, Reno, NV, 89557, USA; Department of Physiology and Cell Biology, University of Nevada, Reno School of Medicine, 1664 North Virginia Street, Reno, NV, 89557, USA; Department of Physiology and Cell Biology, University of Nevada, Reno School of Medicine, 1664 North Virginia Street, Reno, NV, 89557, USA; Department of Physiology and Cell Biology, University of Nevada, Reno School of Medicine, 1664 North Virginia Street, Reno, NV, 89557, USA; Department of Physiology and Cell Biology, University of Nevada, Reno School of Medicine, 1664 North Virginia Street, Reno, NV, 89557, USA; Department of Physiology and Cell Biology, University of Nevada, Reno School of Medicine, 1664 North Virginia Street, Reno, NV, 89557, USA; Department of Physiology and Cell Biology, University of Nevada, Reno School of Medicine, 1664 North Virginia Street, Reno, NV, 89557, USA; Department of Physiology and Cell Biology, University of Nevada, Reno School of Medicine, 1664 North Virginia Street, Reno, NV, 89557, USA; Department of Life and Health Science, Dundalk Institute of Technology, Dublin Road, Dundalk, Co. Louth, A91 K584, Ireland; Department of Physiology and Cell Biology, University of Nevada, Reno School of Medicine, 1664 North Virginia Street, Reno, NV, 89557, USA

**Keywords:** renal pelvis, smooth muscle, upper urinary tract, Anoctamin-1, interstitial cells, genetically encoded calcium indicator

## Abstract

The process of urine removal from the kidney occurs via the renal pelvis (RP). The RP demarcates the beginning of the upper urinary tract and is endowed with smooth muscle cells. Along the RP, organized contraction of smooth muscle cells generates the force required to move urine boluses toward the ureters and bladder. This process is mediated by specialized pacemaker cells that are highly expressed in the proximal RP that generate spontaneous rhythmic electrical activity to drive smooth muscle depolarization. The mechanisms by which peristaltic contractions propagate from the proximal to distal RP are not fully understood. In this study, we utilized a transgenic mouse that expresses the genetically encoded Ca^2+^ indicator, GCaMP3, under a myosin heavy chain promotor to visualize spreading peristaltic contractions in high spatial detail. Using this approach, we discovered variable effects of L-type Ca^2+^ channel antagonists on contraction parameters. Inhibition of T-type Ca^2+^ channels reduced the frequency and propagation distance of contractions. Similarly, antagonizing Ca^2+^-activated Cl^−^ channels or altering the transmembrane Cl^−^ gradient decreased contractile frequency and significantly inhibited peristaltic propagation. These data suggest that voltage-gated Ca^2+^ channels are important determinants of contraction initiation and maintain the fidelity of peristalsis as the spreading contraction moves further toward the ureter. Recruitment of Ca^2+^-activated Cl^−^ channels, likely Anoctamin-1, and T-type Ca^2+^ channels are required for efficiently conducting the depolarizing current throughout the length of the RP. These mechanisms are necessary for the efficient removal of urine from the kidney.

## Graphical Abstract

**Graphical Abstract:**
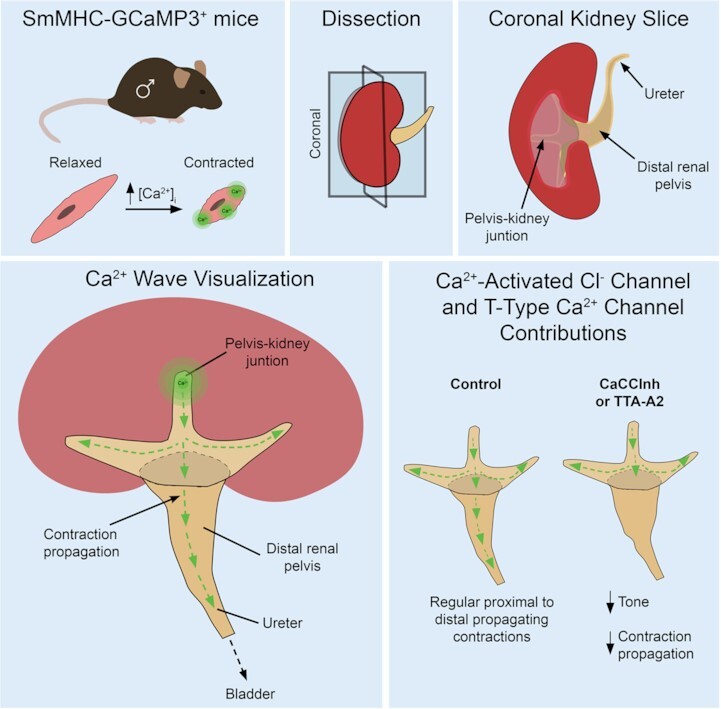

## Introduction

The process of actively transporting urine from the kidneys is vital. After ultrafiltration of the blood in the nephrons, concentrated urine flows into the upper urinary tract via the renal papilla. If urine is left to accumulate in the kidney, hydronephrosis can occur, characterized by a buildup of fluid and eventual renal failure. The renal pelvis (RP) is central to the process of exporting urine from the kidney. As urine enters the RP from the renal papilla, it flows through a connected series of minor and major calyces, and peristaltic contractions of smooth muscle cells that line the wall of the RP pump urine into the ureters toward the bladder^1^. Contractions of the muscle are resistant to tetrodotoxin, guanethidine, and atropine^2–4^, suggesting that the initiation and propagation of the peristaltic contractions are myogenic. However, the definition of “myogenic” has expanded, such that pacemaker activity and muscle contractions appear to depend on at least two specialized populations of cells ^5–9^. The local pacemaker cells have been termed atypical smooth muscle cells^1,5^,^7–17^. Atypical smooth muscle cells are morphologically and electrically distinct from “typical” smooth muscle cells that drive pyeloureteral contractions^5^. Atypical smooth muscle cells densely populate the pelvis–kidney junction (PKJ), where peristaltic contractions originate^13^. Recent evidence suggests that atypical smooth muscle cells possess fibroblast markers and express the Ca^2+^-activated Cl^−^ channel (CaCC), *Ano1*^12,18^, a channel that is also involved in the pacemaker activity of other visceral smooth muscle organs (e.g., gastrointestinal tract)^19^. As a result of this finding, we now refer to atypical smooth muscle cells as platelet-derived growth factor receptor alpha-positive interstitial cells type 1 (PIC1). Changes in ANO1 expression is also implicated in disease. For example, in human ureteropelvic junction samples, decreased ANO1 expression is observed during ureteropelvic junction (UPJ) obstruction^20^. However, the mechanisms by which ANO1 channels participate in the peristaltic contractions of the RP are not fully understood.

Peristaltic contractions in the RP are initiated in typical smooth muscle cells in the proximal region and propagate distally for efficient transport of urine from the kidney^21–23^. In unicalyceal mammals (i.e., mice, rats, and guinea pigs), the rate of spontaneous electrical events is higher in the proximal region than in the distal RP^7^. Previous studies suggested that smooth muscle cells in the distal RP express tetraethylammonium and 4-aminopyridine sensitive K^+^ channels that affected the activation of L-type Ca^2+^ channels during propagating depolarizations^24^. It was also reported that the frequency of spontaneous electrical depolarizations is negatively correlated with the number of atypical smooth muscle cells along the RP from proximal to distal^13^. Several ion channels are proposed to be involved in propagating contractions, and expression analysis of murine RP showed that, in addition to *Ano1*, genes encoding voltage-gated Ca^2+^ channels, including L-type Ca^2+^ channels (Ca_V_1.2 and Ca_V_1.3) and T-type Ca^2+^ channels (Ca_V_3.1 and Ca_V_3.2), are expressed^18,25^. Other groups have suggested that hyperpolarization-activated cyclic nucleotide-gated (HCN) channels initiate peristaltic contractions^25–28^. Contributions of these ion channels to muscle strip contractions have been investigated^27,28^, but few studies have attempted to understand the functional role of specific conductances in the contractile waves that propagate from the proximal RP to the ureter.

In the present study, we developed a model to monitor propagating peristaltic contractions in the RP. We utilized mice that conditionally express the genetically encoded Ca^2+^ indicator, GCaMP3 in renal pelvic smooth muscle cells, making it possible to monitor Ca^2+^ transients that activate smooth muscle cells via voltage-dependent Ca^2+^ channels. This model makes it possible to visualize propagating peristaltic contractions, initiated at the PKJ and traversing the RP toward the ureter with high spatial and temporal resolution. We used this model to test our hypothesis that peristaltic contractions are initiated in the PKJ and propagate with relatively high efficiency to the distal pelvis and ureter and that the fidelity of propagation is provided by activation of specific voltage-dependent Ca^2+^ channel conductances and recruitment of ANO1 channels. The approach that we developed during this study allows accurate mapping of propagating Ca^2+^ waves underlying peristalsis. We found that T-type Ca^2+^ channels and the Ca^2+^-activated Cl^−^ channel ANO1 are important determinants of peristaltic propagation distance in the RP.

## Materials and Methods

### Ethical Approval

Mice were maintained and experiments were performed in accordance with the National Institutes of Health Guide for the Care and Use of Laboratory Animals, and the Institutional Animal Care and Use Committee at the University of Nevada, Reno, NV, approved experimental protocols. Mice were fed *ad libitum*, had free access to water, and were provided with appropriate enrichment. Mice were anesthetized by inhalation of 3%–4% isoflurane in oxygen. After induction of deep anesthesia (Plane III, analgesic) had been validated by loss of toe and/or tail pinch reflex, mice were euthanized by cervical dislocation.

### Mouse Strains

Female *GCaMP3*^lox/+^ mice (B6.129S-*Gt(ROSA)26Sor^tm38(CAG-GCaMP3)Hze^*/J) were crossed with male smMHC-iCre mice (B6.FVB-Tg Myh11-cre/ER^T2^) to generate smMHC^GCaMP3/+^ offspring (mice will be referred to as SMC-GCaMP). Both *GCaMP3*^lox/+^ and smMHC-iCre transgenic strains were purchased from The Jackson Laboratory. Since Cre recombinase expression is driven from the Y chromosome in B6.FVB-Tg Myh11-cre/ER^T2^ mice, only male mice underwent Cre recombination. Therefore, no female SMC-GCaMP3 mice were used in this study. Male SMC-GCaMP3 mice were used between 12 and 20 weeks of age. Male and female wild-type mice (i.e., C57BL/6J;  Jackson Laboratory), aged 8–20 weeks, were used for contractile experiments.

### Tamoxifen Administration and Cre Recombinase Activation

Expression of GCaMP3 was induced in smMHC^+^ cells by injection of tamoxifen into SMC-GCaMP3 mice at > 8 weeks of age (2 mg for three consecutive days). Tamoxifen (Sigma) was dissolved in ethanol (Phamco-Aaper, 200 proof) and vortexed for 20 min. Safflower oil was added, followed by sonication for 30 min to bring the final concentration of tamoxifen to 20 mg/mL. A total of 2 mg of tamoxifen was administered IP for three consecutive days for Cre recombinase induction. Successful induction was confirmed by genotyping 10 days after the initial tamoxifen injection. Once Cre recombinase expression had been confirmed, tissues from the mice were used for fluorescent imaging experiments.

### Tissue Preparation

**Figure 1.**
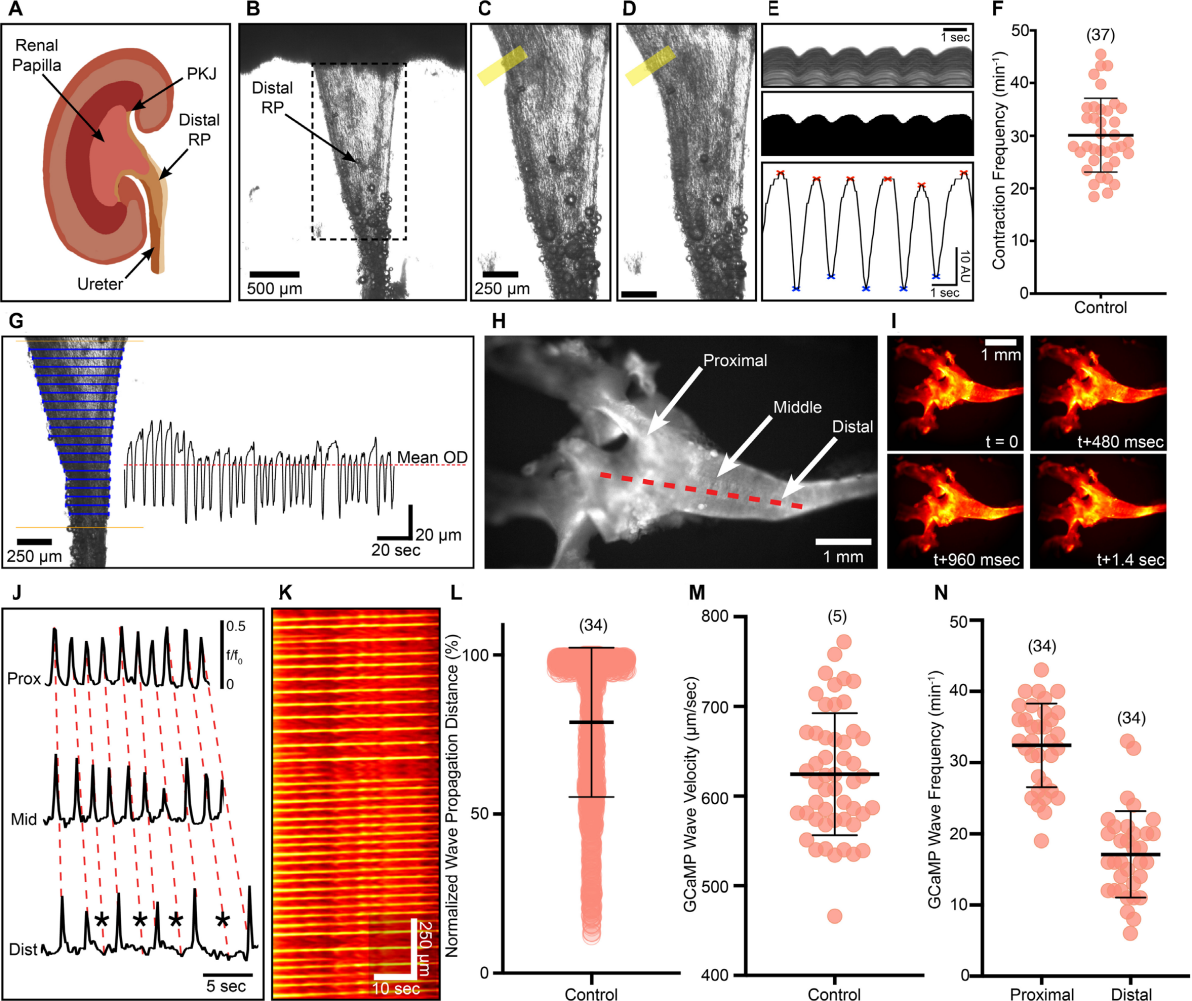
Overview of experimental procedures used to measure propagating renal pelvis (RP) peristalsis. (A) A coronal section of the murine kidney showing the position of the inner RP and important anatomical locations including the renal papilla, pelvis–kidney junction (PKJ), distal RP, and ureter. (B) Representative image showing an exposed mid- and distal RP dissected of adipose tissue that would otherwise obscure a clear view of the RP. Dashed rectangle represents area of the RP that is analyzed during contractions measurements. (C) Zoom of the RP as in panel (B). Yellow rectangle represents where a 50-pixel line-scan is placed over the outer edge of the middle RP region. Panel (D) shows a contractile wave that has propagated past the yellow rectangle, causing the edge of the tissue to move across the line-scan. (E) Top: line-scans from panels (C) and (D; yellow rectangle) are converted into a spatio-temporal map that shows the movement of the tissue across the line. Peaks and valleys can be visualized over time. Middle: image data is thresholded and a line-scan traced through the entire image. Bottom: this generates a 2D line trace, where trace maxima and minima can be detected automatically. (G) Example of outer edge detection in Vasotracker software (blue lines fixed between tissue). Lines are drawn between boundaries (gold lines). The mean outer diameter (OD) can be calculated by averaging the 2D line trace generated from all scan-lines on the tissue by finding the mean of all values over time (red-dashed line). (H) Example image of a SMC-GCaMP3-expressing RP tissue with anatomical ROIs including the proximal, middle, and distal RP. The red-dashed line indicates the placement of a line-scan used to generate a spatio-temporal map to measure the length of propagating Ca^2+^ waves across the RP from the proximal to distal region. (I) Images of SMC-GCaMP3 preparation as in panel (H) showing the propagation of a single Ca^2+^ wave from proximal to distal RP over time (1.4 sec total). Image color is coded for Ca^2+^ fluorescence intensity. (J) Representative Ca^2+^ wave line traces from SMC-GCaMP3^+^ proximal (Prox), middle (Mid), and distal (Dist) RP. Red-dashed lines trace the propagation of a Ca^2+^ wave originating in the proximal RP toward the distal RP. Occasionally, Ca^2+^ waves that emanate from the proximal region do not pass into the distal region (asterisks). (K) Spatio-temporal map of the line-scan placed on the SMC-GCaMP3^+^ RP preparation in panel (H). Map is color coded for Ca^2+^ fluorescence intensity. (L) Normalized Ca^2+^ wave propagation distance (%) plot under control conditions showing the trend for SMC-GCaMP3^+^ preparations (*N* = 34, number of data points = 1619). (M) SMC-GCaMP3^+^ Ca^2+^ wave velocity (µm s^−1^) under control conditions (*N* = 5, number of data points = 50). (N) SMC-GCaMP3^+^ Ca^2+^ wave frequency (min^−1^) under control conditions measured in the proximal and distal RP (*N* = 34 for each group). Data presented in panels (L) and (M) is presented as mean ± SD.

Kidneys from SMC-GCaMP and wild-type mice were removed and immediately placed into ice-cold Krebs Ringer bicarbonate (Krebs) solution containing (in mM); 120.35 NaCl 5.9 KCl, 15.5 NaHCO_3_, 1.2 Na_2_HPO_4_, 1.2 MgCl_2_, 11.5 glucose, and 2.5 CaCl_2_, and bubbled with a mix of 97% O_2_ and 3% CO_2_. For SMC-GCaMP3 preparations, the RP was dissected from the surrounding parenchymal and adipose tissue. Care was taken during dissection to minimize trauma to the delicate PKJ. For wild-type contraction recordings, adipose tissue was sharply dissected from the distal RP, the renal capsule removed, and the kidney sliced sagittal with a blade to expose the PKJ and proximal RP. The papilla was then removed by cutting its connection with the cortex. An example of this preparation is shown in Figure 1(A) and (B). Wild-type preparations were pinned loosely through the cortex and distal ureter in a sylgard coated 35 mm imaging dish and immersed in ice-cold Krebs solution before equilibration. Tissues were maintained on ice in Krebs solution until imaging for no more than 3 hours. Wild-type and SMC-GCaMP tissues were equilibrated at 36–37°C for 1–2 hours and perfused with Krebs solution at a rate of 2–3 ml min^−1^.

### Wild-type Contraction Recordings

The distal RP and connected proximal ureter were imaged on an inverted Nikon Eclipse Ti microscope with a 2x Nikon objective. Images were acquired using a monochrome camera (effective imaging area: 2500 × 2500 µm; The Imaging Source, Charlotte, NC) and sampled at 20 Hz using IC Image Capture software (The Imaging Source). For online measurements of outer diameter (OD), images were sampled at 3 Hz using custom Vasotracker^29^ software. After a control period, RP preparations were exposed to increasing concentrations of pharmacological compounds (1, 10, 100, 1, and 10 µM) for 10 min followed by a 5-min recording period. Vehicle control experiments were also performed in DMSO with a dose response application calculated from the highest concentration of DMSO used (0.01%).

### Wild-type Contraction Recording Analysis

Time-lapse images were analyzed offline in either Fiji^30^ or Vasotracker software. For Fiji analysis, images were loaded in as AVI using the BioFormats plugin. A line-scan (50 px thickness) was drawn on the distal RP as it exits the kidney hilum and the reslice tool used to generate a spatio-temporal map of the tissue movement over time. The spatio-temporal map was thresholded to remove noise and a Gaussian filter was applied to smooth the trace (to improve maxima and minima peak detection). The entire spatio-temporal map was selected, and a plot profile of the spatio-temporal map was generated. The broadly applicable routines plugin package was used to find peaks (maxima and minima) in the plot profile. A list of detected peaks was generated that included the time point at which the maxima and minima occurred vs. peak height (amplitude). A custom Python script was used to automatically calculate the frequency, amplitude and peak–peak interval variance for each detected peak in the plot profile. Peak amplitudes below a specified threshold (mean–2 SD), were not counted as a detectable event for frequency, peak–peak interval or peak–peak interval variance calculations but were plotted for peak amplitude values. Diameter measurements were acquired using Vasotracker software (both online and offline versions). Multiple lines scan lines (10) were traced on to the RP using the ROI mode to measure the OD for each line (example shown in Figure 1G). An average value for the 10 lines was calculated and plotted as a line trace. For each drug concentration used, the average was calculated of the averaged 10-line trace to generate a mean OD value.

### Measurement of Ca^2+^ Waves in ^GCaMP3/+^smMHC Tissues

Fluorescent Ca^2+^ wave measurements were acquired from the entire RP tissue of SMC-GCaMP mice. Propagating (proximal–distal) Ca^2+^ waves were visualized using an upright Nikon Eclipse E600FN microscope equipped with a 4x Plan Nikon objective lens. Images were acquired using an sCMOS camera (Andor Neo sCMOS, Oxford Instruments, Belfast, UK). Images were sampled between 5 and 16 Hz using Andor Solis software. During image acquisition, the following protocol was used for each experiment: (1) control period 0–10 min, (2) pharmacological intervention period 10–30 min, and (3) washout period in Krebs solution 30–60 min. These time periods were chosen to allow time for drugs to reach specified bath concentrations during constant perfusion.

### Ca^2+^ Imaging Analysis

Propagation of Ca^2+^ waves in intact RP tissues were analyzed using spatio-temporal mapping. 8-bit time-lapse image stacks were converted to 32-bit image formats in Fiji software. For each raw image stack, the mean background intensity was sampled and subtracted from the entire series. To create spatio-temporal maps, line-scans were traced and extended through the entire RP, using either the straight- or segmented-line tool. The spatio-temporal map was then generated by evoking the reslice function. To calibrate spatio-temporal map intensity and generate F/F_0_ values, basal Ca^2+^ fluorescence (F_0_) was sampled and measured in a region of the pelvis during no activity (i.e., no propagating Ca^2+^ wave). The entire time-series was then divided by F_0_ to provide a spatio-temporal map with amplitude expressed as F/F_0_.

### Trace Generation and Wave Frequency

All representative traces for vehicle or pharmacological intervention experiments are derived from 1hour recordings or short-interval recordings of the intact RP at 5 Hz. The substack tool (in Fiji) was used to generate 2-min interval videos of the control period, drug incubation period, and washout period. Proximal renal pelvic SMC-GCaMP traces were generated from line-scans resliced in the proximal region of the pelvis, and distal renal pelvic SMC-GCaMP traces created from line-scans resliced from the distal region of the pelvis. The frequency of proximal and distal Ca^2+^ waves (min^−1^) was calculated from these two regions.

### Propagation Analysis

To measure the distance that Ca^2+^ waves propagated along the RP, 2-min interval substacks from the entire time-lapse were resliced and spatio-temporal maps generated. Spatio-temporal maps were calibrated spatially and temporally. Using the straight-line tool, propagating Ca^2+^ waves were measured manually to derive a cumulative count for the total number of waves (frequency) and distance each Ca^2+^ wave traveled along the length of the entire RP (µm). Measurements were then saved as .txt files and analyzed. A custom Perl script was used to calculate the longest propagating Ca^2+^ wave across the pelvis during each recording. To normalize measurements, every other line from the spatio-temporal map was divided by the longest measurement within the spatio-temporal map.

### Drugs and Solutions

CaCC_inh_-A01 (Tocris), benzbromarone (Tocris), nicardipine (Sigma), TTA-A2 (Alamone), and isradipine (Tocris) were dissolved in DMSO (concentration of DMSO in solution did not exceed 0.01%). ZD7288 (Tocris) was dissolved in water. Furosemide (Tocris) and bumetanide (Tocris) were dissolved in 100% ethanol (ethanol concentration did not exceed 0.01%). Nominal Ca^2+^ solutions (0 mM Ca^2+^ + 1 mM EGTA) contained (in mM): 120.35 NaCl 5.9 KCl, 15.5 NaHCO_3_, 1.2 Na_2_HPO_4_, 1.2 MgCl_2_, 11.5 glucose, 2.5 MgCl_2_, and 1.0 EGTA.

### Statistical Analysis

All figures were plotted using R 4.1.3 (through RStudio) or GraphPad Prism (ver.9). All data are presented as means ± SD Statistical analyses were performed using Student’s paired or unpaired *t*-test where appropriate. *P-*values < .05 are considered statistically significant. *N* refers to the number of animals used.

## Results

Coronal kidney sections with RP attached (Figure 1A and B) were equilibrated for 1 h. During the equilibration period, peristaltic contractions became regular and the majority of contractions propagated the length of the preparation in the field-of-view (Figure 1C and D). However, some contractions did not propagate over the entire length of the RP (Supplemental Video 1). Line scans taken of the middle region of the pelvis (yellow rectangles in Figure 1C and D) show peristaltic contraction occurring at regular intervals (Figure 1E) at an average frequency of 30.11 ± 7.02 contractions min^−1^ (*N* = 37; Figure 1F). The frequency of contractions observed in our study is significantly higher than that in other studies using similar contractile assays^31^. This assay was used to measure responses to the ion channel antagonists tested in this study. The distances traveled by propagating contractions and differences between contraction frequency in the proximal and distal RP were measured using imaging of Ca^2+^ waves in SMC-GCaMP3 preparations (Figure 1H). Under control conditions, Ca^2+^ waves emanated from the most proximal portion of the preparations (in the PKJ) and propagated down to the ureter (Figure 1I). Time-lapse images in Figure 1I represent a propagating Ca^2+^ wave that swept across the RP from the proximal to distal margins. As with measurements of contractions in wild-type preparations, some Ca^2+^ waves did not propagate the full length of the RP and terminated before reaching the distal portion (Figure 1J; asterisks denote Ca^2+^ waves that failed to propagate to the distal end of the RP; Supplemental Video 2). Single pixel line-scans were traced longitudinally to measure the propagation of Ca^2+^ waves (i.e., from proximal to distal ends of the preparations, e.g., red-dotted line in Figure 1H). Spatio-temporal maps were generated from line-scans that represent the propagation of Ca^2+^ waves over time (Figure 1K). Lines within spatio-temporal maps that correlate to propagating Ca^2+^ waves (Figure 1K) were measured manually and normalized to the length of the longest Ca^2+^ wave (i.e., one that spread all the way to the ureter). We observed a large range of Ca^2+^ wave propagation lengths in most preparations studied (22/34 preparations; Figure 1L; mean = 78.89 ± 23.49%, number of values = 1619, *N* = 34). Propagating Ca^2+^ waves traveled at a velocity of 624 ± 68 µm s^−1^ (Figure 1M; number of values = 50, *N* = 5). To assay spontaneous Ca^2+^ transient frequency, we selected regions of interest (ROIs) on the proximal and distal pelvis. The frequency of Ca^2+^ waves decreased between the proximal and distal regions (Figure 1N). This represents a lack of full Ca^2+^ wave propagation through to the ureter (proximal GCaMP3 wave frequency: 32.44 ± 5.86 waves min^−1^ vs. distal GCaMP3 wave frequency: 17.12 ± 6.06 waves min^−1^, *N* = 34 for each region).

**Figure 2.**
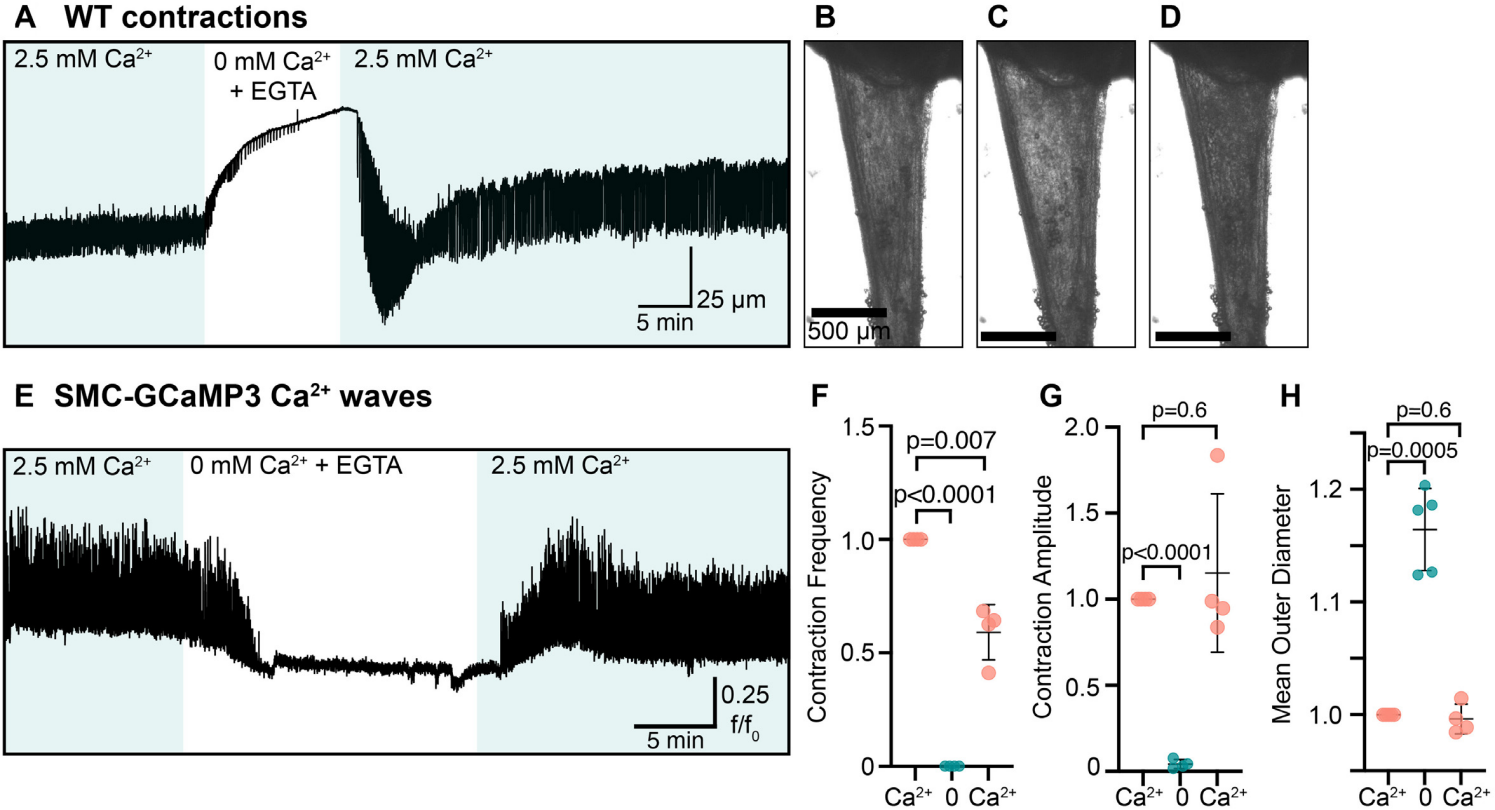
Ca^2+^ is critical for maintaining contraction frequency and RP smooth muscle tone. (A) Representative trace showing WT contractions of the RP in the presence and absence of Ca^2+^ (2.5 mM). Nominal Ca^2+^ solutions contained 1 mM EGTA to buffer residual Ca^2+^. (B) Image of a RP in the presence of 2.5 mM Ca^2+^. (C) Image of the same RP but in the presence of nominal Ca^2+^ (+ 1 mM EGTA). (D) Image of same RP but after 2.5 mM Ca^2+^ is reintroduced into the bathing solution. Scale bars in (C) and (D) are 500 µm. (E) Representative trace showing SMC-GCaMP3 Ca2 + waves in the presence and absence of 2.5 mM Ca^2+^. Nominal Ca^2+^ solution contained 1 mM EGTA to buffer residual Ca^2+^. (F)–(H) Plots showing changes in contraction frequency (F), contraction amplitude (G), and mean outer RP diameter (H) when removing and reintroducing Ca^2+^ from the perfusion solution (*N* = 4 for each panel; data presented as mean ± SD, Student’s paired *t-*test).

When the RP was bathed in Krebs solution containing 2.5 mM CaCl_2_, contractions occurred at regular intervals and tone was maintained (Figure 2A and B). When tissues were bathed in Krebs in the absence of Ca^2+^ (and buffered with EGTA; 1 µM), the RP lost tone (Figure 2A and C). Upon reintroduction of 2.5 mM Ca^2+^ to the bath solution, contractions resumed, and tone was generated (Figure 2A and D). In SMC-GCaMP3 preparations, Ca^2+^ waves became unresolvable in the absence of Ca^2+^ (Figure 2E). Quantification of these experiments (contraction frequency, amplitude, and mean RP OD) are provided in Figure 2F–H.

**Figure 3.**
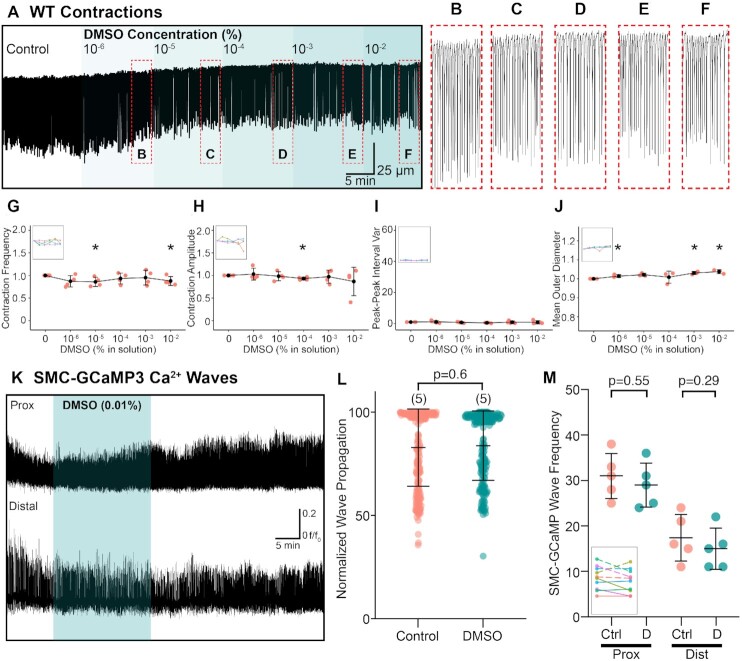
Vehicle DMSO has minimal effects on RP contractions. (A) Representative muscle contraction trace from the RP and the effect of increasing concentrations of DMSO (10^−6^–10^−2^ M). (B)–(F) Expanded contraction trace subset from contraction trace in (A). (G)–(J) Contraction frequency (C), contraction amplitude (D), peak–peak interval variance (E), and mean outer RP diameter (F) vs. DMSO (% in solution) plots with insets showing connected individual experiments (*N* = 4 for each panel; data presented as mean ± SD; values for each concentration are compared against the paired control value; Student’s paired *t-*test). For all plots *P ≤ .05. (K) Time course of Ca^2+^ transients measured from the proximal (Prox) and distal RP in the presence and absence of the highest DMSO concentration (0.01%) used for assays where DMSO is used as solvent. (L) Normalized wave propagation scatter plot for control and DMSO-treated SMC-GCaMP3 RP preparations. Each point represents one Ca^2+^ wave measurement. *N* = 5 for each group. (M) Plot of SMC-GCaMP3 wave frequency sampled from proximal (Prox) and distal (Dist) regions in the presence of control (Ctrl) and DMSO (D; 0.01%) solutions. Inset shows individual experiments from proximal (dotted lines) and distal (solid lines) regions.

The effects of DMSO on RP contractions were also tested. DMSO was the vehicle used for most pharmacological compounds studied (except ZD7288, which was solubilized in deionized H_2_O and furosemide and bumetanide, that were solubilized in ethanol). Example traces from the contraction assay performed on wild-type RP show minimal effects of DMSO even at the highest solvent concentration used (0.01%; Figure 3A–F). Over the course of the experiment on a wild-type RP shown in Figure 3A, contraction frequency tended to wax and wane. Occasionally, contraction frequency significantly changed vs. control conditions (Figure 3G; at 10^−5^ and 10^−2^% DMSO concentration in solution, *N* = 4). However, contraction amplitude was stable throughout the recording period but decreased significantly at 10^−4^% DMSO in solution but recovered thereafter (Figure 3H; *N* = 4). Peak–peak interval variance did not change significantly as a function of DMSO concentration (Figure 3I; *N* = 4), but RP mean diameter tended to fluctuate (Figure 3J; *N* = 4). For SMC-GCaMP3 tissues, representative traces in Figure 3K show little change when in the presence of DMSO (0.01%) in the proximal or distal RP. Normalized Ca^2+^ wave propagation did not change significantly between control and DMSO application (Figure 3L; *P* = .6, control: 82.83 ± 18.73% vs. 0.01% DMSO: 83.74 ± 16.78%, number of control values = 219, number of DMSO values = 197, *N* = 5 for each group). Similarly, SMC-GCaMP3 wave frequency was not significantly different between control and DMSO (Figure 3M) in either the proximal (*P=* .55, *N* = 5 for each group) or distal (*P* = .29, *N* = 5 for each group) RP.

**Figure 4.**
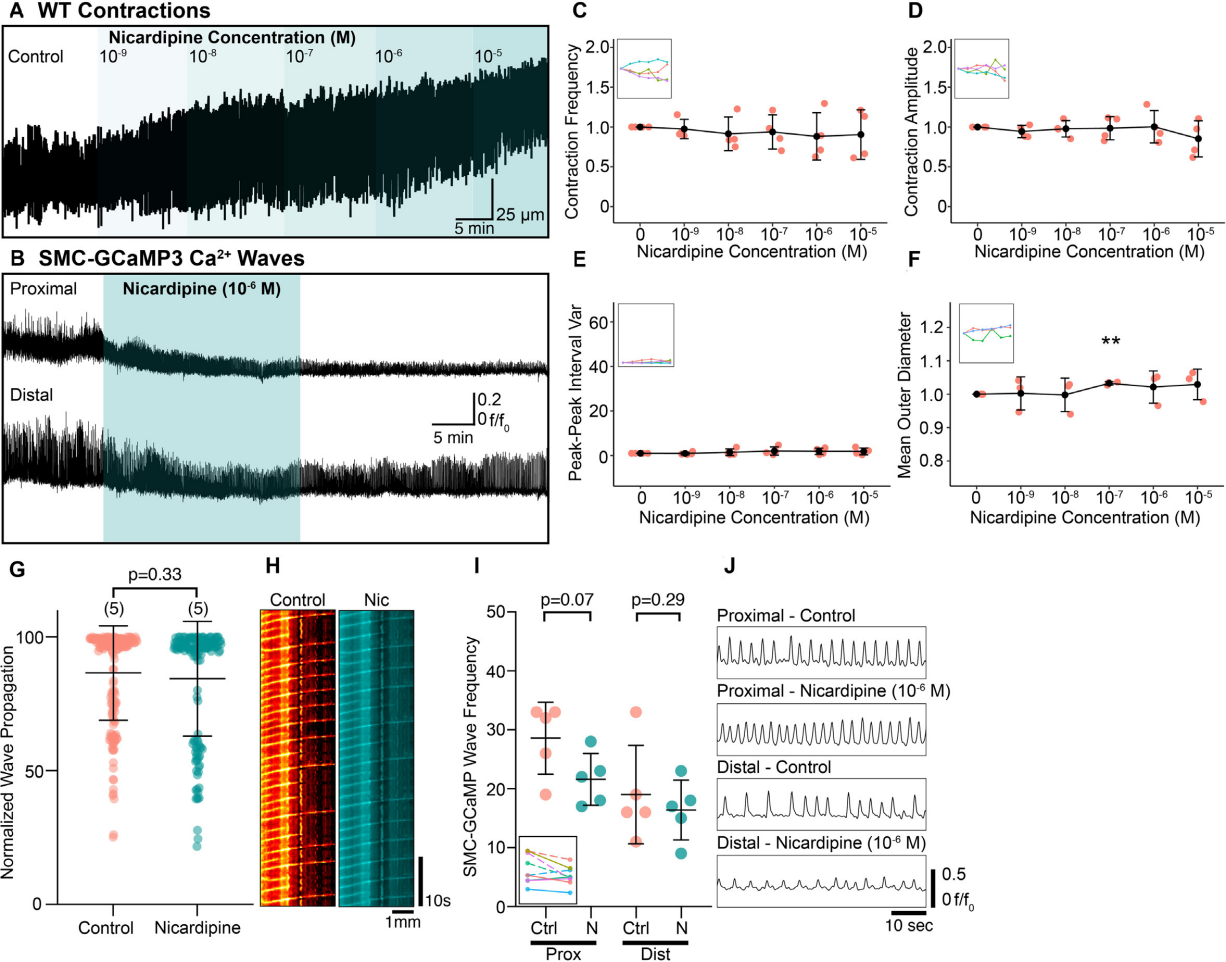
RP contractions are relatively insensitive to L-type Ca^2+^ channel antagonism with nicardipine. (A) Representative contraction trace demonstrating the effect of increasing concentrations of nicardipine (10^−9^–10^−5^ M). (B) Exemplar Ca^2+^ wave traces recorded from the proximal and distal RP in the presence and absence of the nicardipine (10^−6^M). (C)–(F) Contraction frequency (C), contraction amplitude (D), peak–peak interval variance (E), and mean outer RP diameter (F) vs. increasing nicardipine concentration plots with insets showing connected individual experiments (*N* = 4 for panels (C)–(E), *N* = 3 for panel (F); data presented as mean ± SD; values for each concentration are compared against the paired control value; Student’s paired *t*-test). For all plots **P ≤ .01. (G) Normalized wave propagation scatter plot for control and nicardipine (10^−6^ M)-treated SMC-GCaMP3 RP preparations. Each point represents one Ca^2+^ wave measurement. *N* = 5 for each group. (H) Spatio-temporal maps showing the Ca^2+^ waves propagating the length of RP under control and nicardipine conditions. (I) SMC-GCaMP3 wave frequency plot measured from proximal (Prox) and distal (Dist) regions in the presence of control (Ctrl) and nicardipine (N; 10^−6^ M) solutions. Inset shows individual experiments from proximal (dotted lines) and distal (solid lines) regions. (J) Representative line traces of Ca^2+^ waves sampled from proximal or distal regions treated with control or nicardipine (10^−6^ M) solutions. Traces between conditions are acquired from the same regions.

As peristaltic contractions involve excitation–contraction coupling in smooth muscle cells, we investigated antagonists of Ca^2+^ channels that have been associated either with activation of SMCs or pacemaker functions in other visceral smooth muscle cells to characterize the contributions of L-type and T-type Ca^2+^ channels in peristaltic contractions. We found that wild-type RP tissues continued to contract during the highest drug doses of nicardipine tested (10^−4^–10^−5^ M), suggesting a relatively high degree of resistance toward this L-type Ca^2+^ channel antagonist (Figure 4A). Similarly, GCaMP3 Ca^2+^ signals from proximal and distal pelvis were not inhibited significantly by nicardipine (Figure 4B). This was also consistent with wild-type tissue experiments, as mean contraction frequency did not significantly change over time (Figure 4C). Contraction amplitude was also stable despite increasing doses of nicardipine and were not significantly different vs. control conditions (Figure 4D). Peak–peak interval variance did not significantly change compared to control, but average RP diameter increased at 10^−7^ M, but decreased back to baseline at higher concentrations (Figure 4E and F). Similarly, nicardipine (10^−6^ M) did not significantly affect normalized Ca^2+^ wave propagation distance (Figure 4G; *P* = .33, control: 86.55 ± 17.65% vs. 10^−6^ M nicardipine: 84.39 ± 21.43%, number of control values = 175, number of nicardipine values = 136, *N* = 5 for each group). Example spatio-temporal maps for propagating Ca^2+^ waves are shown in Figure 4H. There was no significant difference between proximal and distal GCaMP3 wave frequency (Figure 4I) in either the proximal (*P* = .07, *N* = 5 for each group) or distal (*P* = .29, *N* = 5 for each group) RP when exposed to nicardipine (10^−6^ M). Example traces from a SMC-GCaMP3 preparation showed that whilst Ca^2+^ wave frequency remained constant, Ca^2+^ wave amplitude in the distal RP was reduced by nicardipine (10^−6^ M). These observations reinforce previous reports that the effects of dihydropyridines are highly variable in the murine RP^7^.

**Figure 5.**
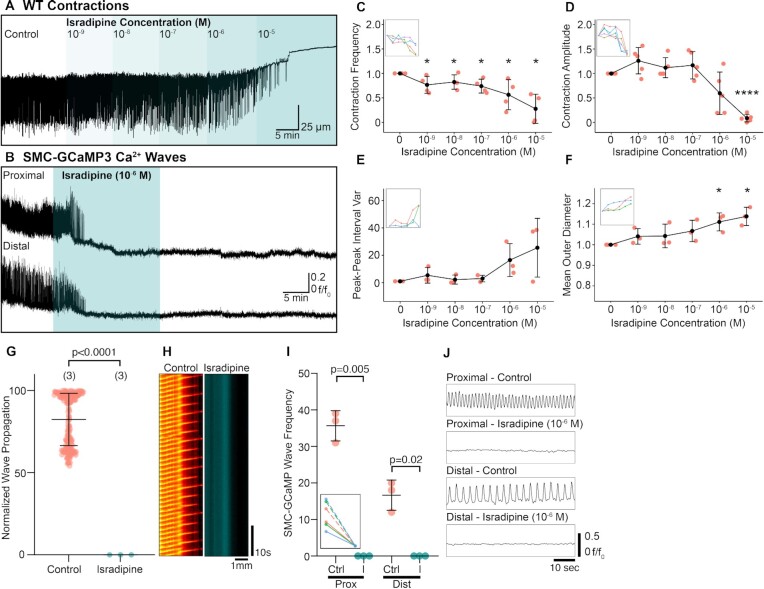
Isradipine rapidly abolishes contractile activity and diminishes Ca^2+^ waves propagating the RP. (A) Representative WT contraction trace demonstrating the effect of increasing concentrations of isradipine (10^−9^–10^−5^M). (B) Representative Ca^2+^ wave traces recorded from the proximal and distal RP transiently treated with isradipine (10^−6^ M). (C)–(F) Contraction frequency (C), contraction amplitude (D), peak–peak interval variance (E), and mean outer RP diameter (F) vs. increasing isradipine concentration plots with insets showing connected individual experiments (*N* = 4 for panel (C), *N* = 5 for panel (D), *N* = 3 for panel (E), and *N* = 3 for panel (F); data presented as mean ± SD; values for each concentration are compared against the paired control value; Student’s paired *t*-test). For all plots *P ≤ .05 and ****P ≤ .0001. (G) Normalized wave propagation plot for control and isradipine (10^−6^M)-treated SMC-GCaMP3 preparations. Each point represents one Ca^2+^ wave measurement. *N* = 3 for each group. (H) Exemplar spatio-temporal maps showing Ca^2+^ waves propagating the length of RP in preparations incubated in control and isradipine solutions. (I) SMC-GCaMP3 Ca^2+^ wave frequency plot measured from proximal (Prox) and distal (Dist) regions in the presence of control (Ctrl) and isradipine (I; 10^−6^ M). Inset shows individual experiments from proximal (dotted lines) and distal (solid lines) regions. (J) Representative line traces of Ca^2+^ waves sampled from proximal or distal regions treated with control or isradipine (10^−6^ M) solutions. Traces between conditions are sampled from the same region on the RP.

Another dihydropyridine, isradipine, had more significant effects on the frequency of contractions in wild-type and SMC-GCaMP3 preparations (Figure 5A and B). In wild-type preparations isradipine (10^−9^ and 10^−5^ M) reduced contraction frequency (Figure 5C). Contraction frequency and amplitude were significantly reduced at 10^−5^ M (Figure 5C and D). In SMC-GCaMP3 preparations, 10^−6^ M was sufficient to inhibit resolution of Ca^2+^ waves in the proximal and distal RP within 5 min of application (Figure 5B). Peak–peak interval variance also significantly increased (Figure 5E), suggesting any remaining contractions at higher isradipine doses (10^−6^–10^−5^ M) were not rhythmic. RP diameter significantly increased at 10^−6^–10^−5^ M isradipine, suggesting a loss of tone (Figure 5F). Since Ca^2+^ waves were not resolved at 10^−6^ M, propagation of Ca^2+^ waves were also significantly inhibited (Figure 5G; *P*< .0001, control: 82.39 ± 15.9% vs. 10^−6^ M isradipine: 0 ± 0%, number of control values = 195, number of isradipine values = 3, *N* = 3 for each group) and Ca^2+^ waves were completely absent in spatio-temporal maps (Figure 5H). Isradipine (10^−6^ M) also inhibited resolution of Ca^2+^ waves in the proximal (*P* = .005, *N* = 3 for each group) and distal (*P* = .02, *N* = 3 for each group) RP (Figure 5I). Example Ca^2+^ wave traces shown in Figure 5J show a total loss of resolvable Ca^2+^ wave activity in the proximal and distal RP during isradipine incubation.

**Figure 6.**
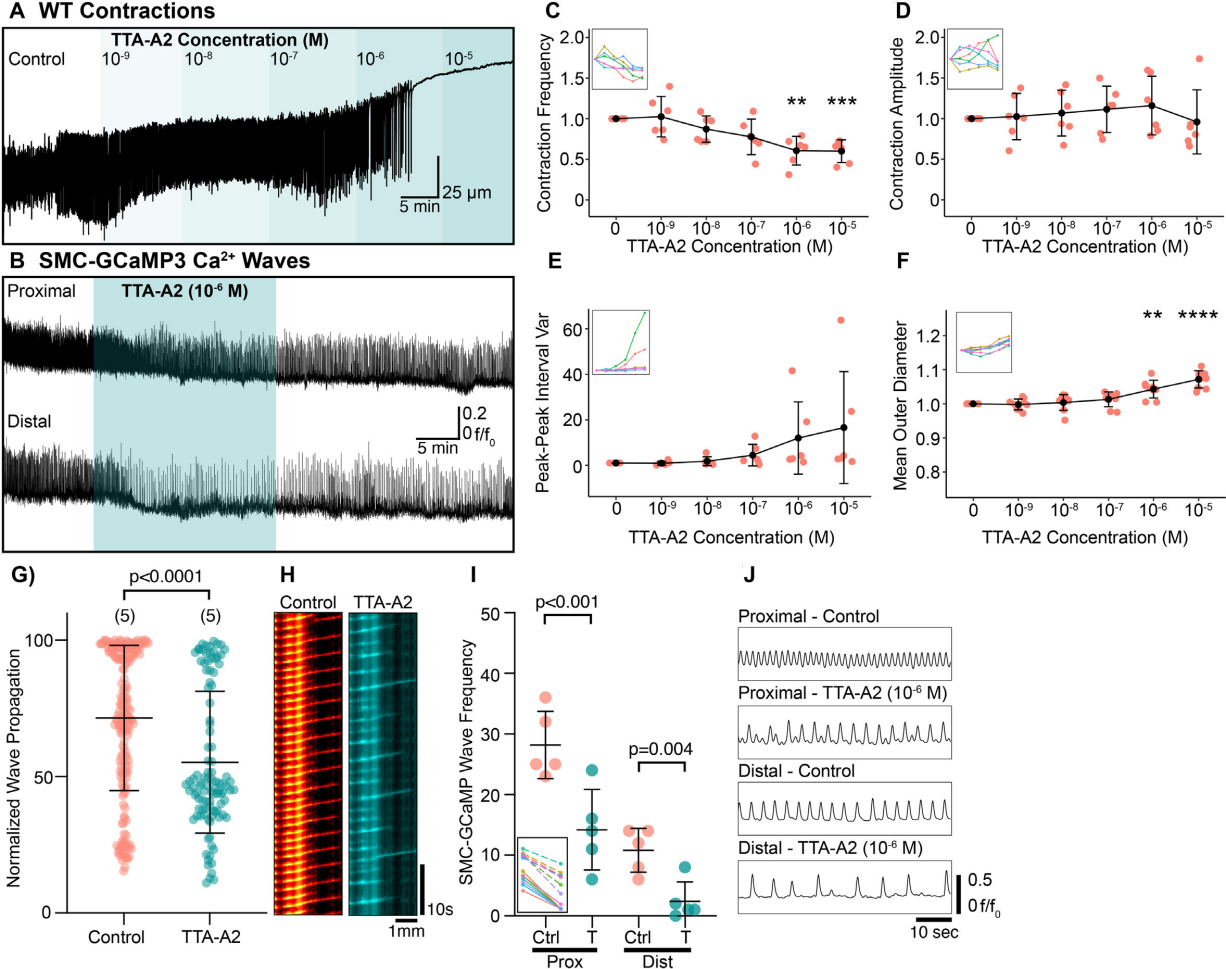
Inhibition of T-type Ca^2+^ channels with TTA-A2 reduces the contractile propagation distance from proximal to distal regions. (A) Exemplar WT contraction trace demonstrating the effect of increasing concentrations of TTA-A2 (10^−9^–10^−5^ M). (B) Ca^2+^ wave traces derived from the proximal and distal RP transiently treated with TTA-A2 (10^−6^ M). (C)–(F) Contraction frequency (C), contraction amplitude (D), peak–peak interval variance (E), and mean outer RP diameter (F) vs. increasing TTA-A2 concentration plots with insets showing connected individual experiments (*N* = 6 for each panel; data presented as mean ± SD; values for each concentration are compared against the paired control value; Student’s paired *t*-test). For all plots **P ≤ .01 and ****P ≤ .0001. (G) Normalized wave propagation plot for control and TTA-A2 (10^−6^ M)-treated SMC-GCaMP3 preparations. Each point represents one Ca^2+^ wave measurement. *N* = 5 for each group. (H) Exemplar spatio-temporal maps showing Ca^2+^ waves propagating the length of RP in preparations incubated in control and TTA-A2 solutions. (I) SMC-GCaMP3 Ca^2+^ wave frequency plot measured from proximal (Prox) and distal (Dist) regions in the presence of control (Ctrl) and TTA-A2 (I; 10^−6^ M). Inset shows individual experiments from proximal (dotted lines) and distal (solid lines) regions. (J) Representative line traces of Ca^2+^ waves sampled from proximal or distal regions treated with control or TTA-A2 (10^−6^ M) solutions. Traces between conditions are sampled from the same region on the RP.

Contributions of T-type Ca^2+^ channels to peristaltic contractions were also tested by exposing preparations to increasing doses of TTA-A2, a potent inhibitor of Ca_V_3.1 and Ca_V_3.2 channels^32,33^. At 10^−6^ M, TTA-A2, reduced contraction frequency in wild-type preparation and completely abolished contractions at 10^−5^ M (Figure 6A). In SMC-GCaMP3 experiments, 10^−6^ M reduced the frequency of proximal and distal Ca^2+^ wave frequency (Figure 6B). Contraction frequency was significantly reduced by TTA-A2 (10^−6^–10^−5^ M; Figure 6C), whereas contraction amplitude did not change significantly *vs*. control (Figure 6D). TTA-A2 (10^−6^ M) increased the peak-to-peak interval variance (Figure 6E), suggesting that TTA-A2 affected pacemaker regularity. RP diameter also increased significantly between 10^−6^ and 10^−5^ M (Figure 6F), suggesting that tone was reduced at concentrations that had significant effects on contractile frequency. In SMC-GCaMP3 preparations, TTA-A2 (10^−6^ M) significantly reduced the propagation distance of Ca^2+^ waves (Figure 6G; *P* < .0001, control: 71.47 ± 26.57% vs. 10^−6^ M TTA-A2: 55.25 ± 26.01%, number of control values = 198, number of TTA-A2 values = 104, *N* = 5 for each group). An example spatio-temporal map in Figure 6H illustrates a reduction in the number of Ca^2+^ waves propagating toward the distal pelvis in the presence of 10^−6^ M TTA-A2. In addition to a reduction in propagating contractions, TTA-A2 significantly decreased the frequency of SMC-GCaMP3 Ca^2+^ waves in the proximal (*P*< .001, *N* = 5 for each group) and distal (*P* = .004, *N* = 5 for each group) RP (Figure 6I). Figure 6J shows example Ca^2+^ wave traces from the proximal and distal pelvis and a reduction in the frequency of Ca^2+^ waves.

**Figure 7.**
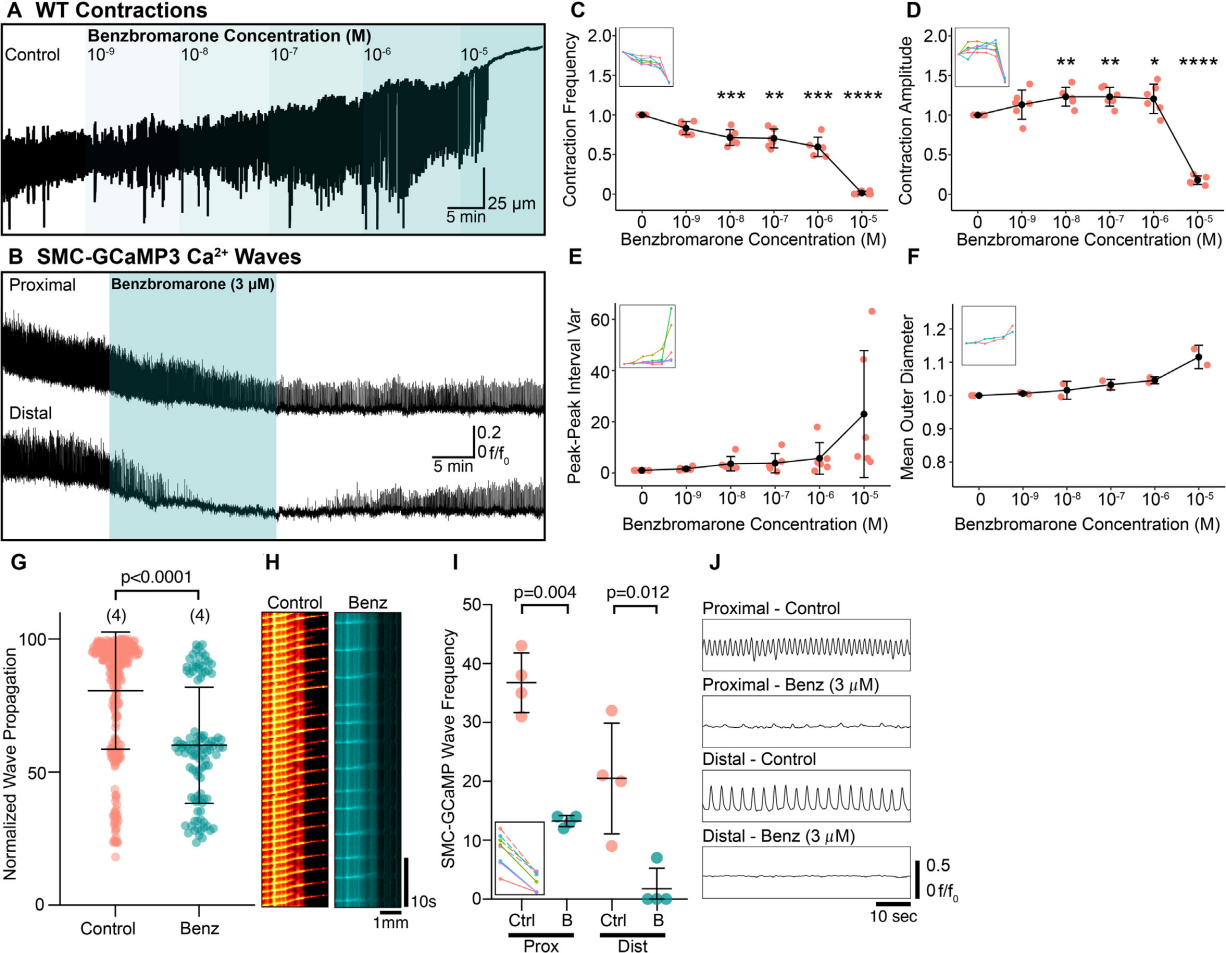
The Ca^2+^-activated Cl^−^ channel antagonist, benzbromarone attenuates peristaltic contraction propagation distance. (A) Representative WT contraction trace demonstrating the effect of increasing concentrations of benzbromarone (10^−9^–10^−5^ M). (B) Ca^2+^ wave traces derived from the proximal and distal RP transiently treated with benzbromarone (3 µM). (C)–(F) Contraction frequency (C), contraction amplitude (D), peak–peak interval variance (E), and mean outer RP diameter (F) vs. increasing benzbromarone concentration plots with insets showing connected individual experiments (*N* = 6 for panels (C)–(E), *N* = 2 for panel (F); data presented as mean ± SD; values for each concentration are compared against the paired control value; Student’s paired *t*-test). For all plots *P ≤ .05, **P ≤ .01, ***P ≤ .001, and ****P ≤ .0001. (G) Normalized wave propagation plot for control and benzbromarone (3 µM)-treated SMC-GCaMP3 preparations. Each point represents one Ca^2+^ wave measurement. *N* = 5 for each group. (H) Exemplar spatio-temporal maps showing Ca^2+^ waves propagating the length of RP in preparations incubated in control and benzbromarone solutions. (I) SMC-GCaMP3 Ca^2+^ wave frequency plot measured from proximal (Prox) and distal (Dist) regions in the presence of control (Ctrl) and benzbromarone (B; 3 µM). Inset shows individual experiments from proximal (dotted lines) and distal (solid lines) regions. (J) Representative line traces of Ca^2+^ waves sampled from proximal or distal regions treated with control or benzbromarone (Benz; 3 µM) solutions. Traces between conditions are sampled from the same region on the RP.

We previously demonstrated that PICs exclusively express ANO1 in the murine RP, suggesting that CaCCs might be a contributing conductance for pacemaker activity driving or supporting peristaltic contractions^18^. Therefore, we tested the effects of two ANO1 antagonists, benzbromarone and CaCC_Inh_-A01, on peristaltic contractions. Benzbromarone (10^−5^ M), abolished RP contractions (Figure 7A and C). Between 10^−9^ and 10^−6^ M, benzbromarone decreased the frequency of contractions significantly (Figure 7C). However, contractile amplitude increased significantly between 10^−8^ and 10^−6^ M and declined at 10^−5^ M (Figure 7D). At 10^−5^ M benzbromarone, peak–peak interval variance increased, but RP mean diameter did not change significantly (Figure 7E and F). Benzbromarone (3 µM), reduced Ca^2+^ wave frequency, particularly in the distal RP (Figure 7B). The propagation distance of Ca^2+^ waves was reduced significantly in the presence of 3 µM benzbromarone (Figure 7G; *P*< .0001, control: 80.66 ± 22.00% vs. 3 µM benzbromarone: 60.12 ± 21.83%, number of control values = 290, number of benzbromarone values = 105, *N* = 4 for each group). An example spatio-temporal map in Figure 7H shows a preparation in which Ca^2+^ waves failed to propagate down the length of the RP in the presence of benzbromarone (3 µM). This compound also reduced the frequency of Ca^2+^ waves in the proximal (*P* = .004, *N* = 4 for each group) and distal (*P* = .012, *N* = 4 for each group) RP (Figure 7I). Traces showing one example of Ca^2+^ waves in the proximal and distal RP illustrate the reduction in frequency in the proximal pelvis and loss of resolvable Ca^2+^ waves in the distal pelvis (Figure 7J).

**Figure 8.**
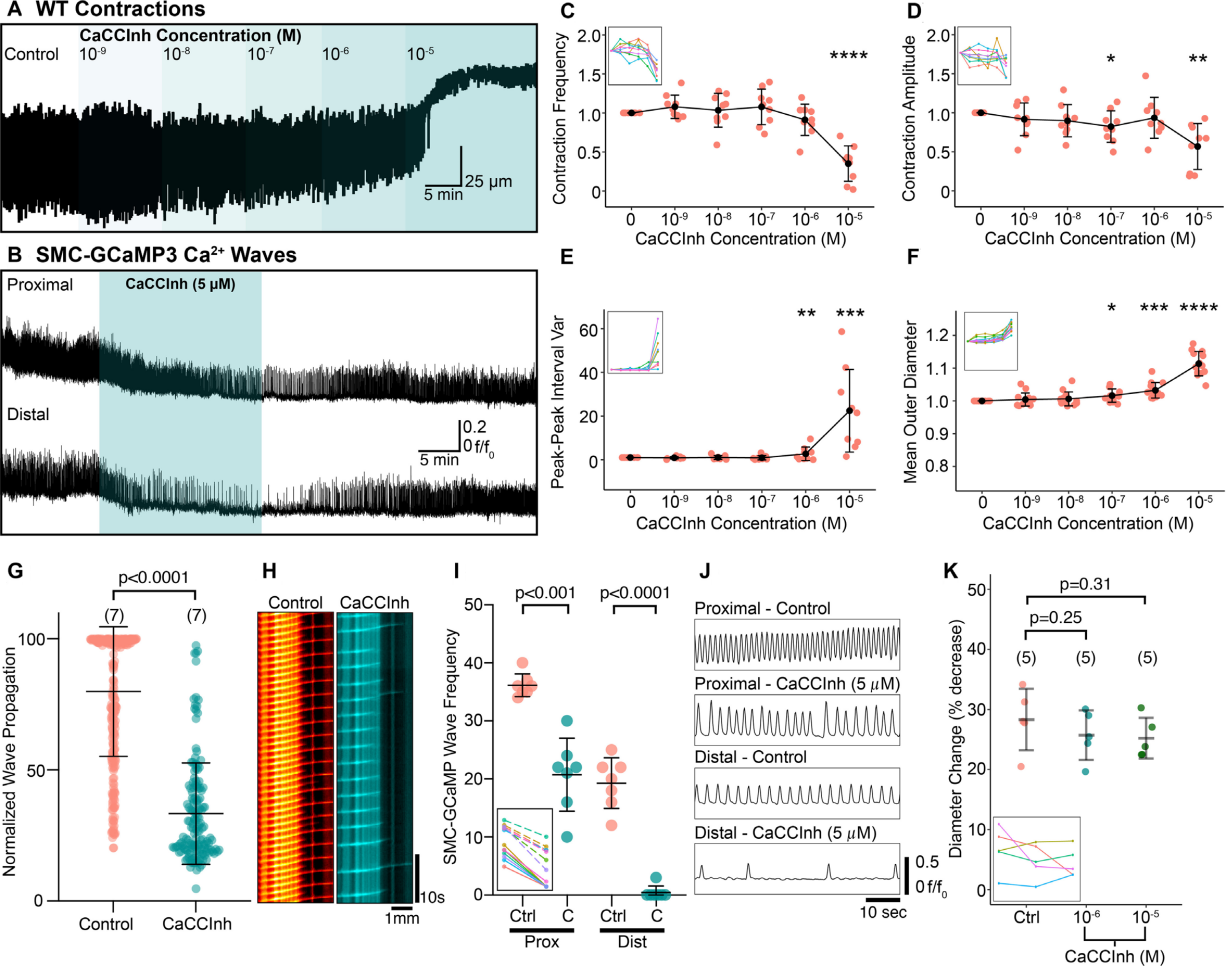
The small-molecule Ca^2+^-activated Cl^−^ channel/TMEM16A antagonist, CaCCInh-A01 potently reduces the propagation of Ca^2+^ waves from proximal to distal RP. (A) Representative WT contraction trace demonstrating the effect of increasing concentrations of CaCCInh-A01 (10^−9^–10^−5^ M). (B) Ca^2+^ wave traces derived from the proximal and distal RP transiently treated with CaCCInh-A01 (5 µM). (C)–(F) Contraction frequency (C), contraction amplitude (D), peak–peak interval variance (E), and mean outer RP diameter (F) vs. increasing CaCCInh-A01 concentration plots with insets showing connected individual experiments (*N* = 9 for panels (C)–(E), *N* = 13 for panel (F); data presented as mean ± SD; values for each concentration are compared against the paired control value; Student’s paired *t*-test). For all plots *P ≤ .05, **P ≤ .01, ***P ≤ .001, and ****P ≤ .0001. (G) Normalized wave propagation plot for control and CaCCInh-A01 (5 µM)-treated SMC-GCaMP3 preparations. Each point represents one Ca^2+^ wave measurement. *N* = 7 for each group. (H) Exemplar spatio-temporal maps showing Ca^2+^ waves propagating the length of RP in preparations incubated in control and CaCCInh-A01 solutions. (I) SMC-GCaMP3 Ca^2+^ wave frequency plot measured from proximal (Prox) and distal (Dist) regions in the presence of control (Ctrl) and CaCCInh-A01 (C; 5 µM). Inset shows individual experiments from proximal (dotted lines) and distal (solid lines) regions. (J) Representative line traces of Ca^2+^ waves sampled from proximal or distal regions treated with control or CaCCInh-A01 (CaCCInh; 5 µM) solutions. Traces between conditions are sampled from the same region on the RP. (K) % decrease in OD of the RP when exposed to 60 mM KCl^−^ under control (Ctrl) conditions and in the presence of 10^−6^ M and 10^−5^ M CaCCInh-A01, *N* = 5 for each group.

**Figure 9.**
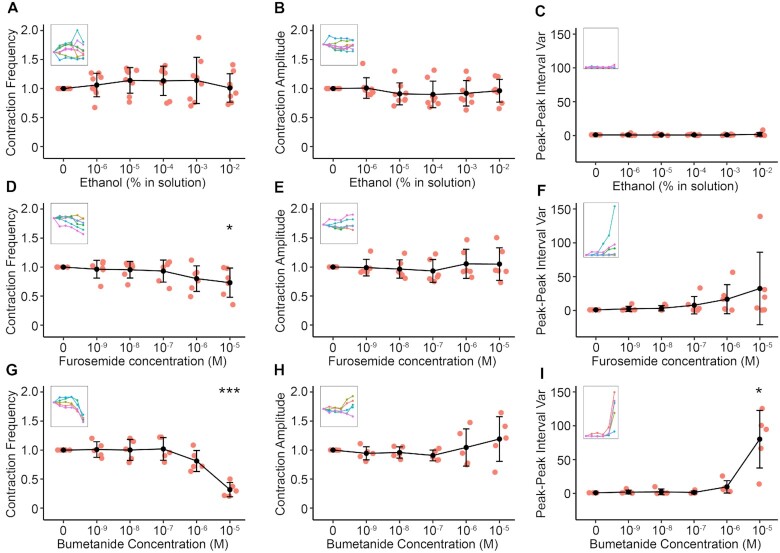
Disrupted chloride gradient with Na-K-2Cl (NKCC) inhibition decreases contraction frequency and causes irregularity. (A)–(C) Contraction frequency (A), contraction amplitude (B) and peak–peak interval variance (C) vs. increasing ethanol concentration plots with insets showing connected individual experiments (*N* = 8; data presented as mean ± SD; values for each concentration are compared against the paired control value; Student'’s paired *t*-test). (D–F) Contraction frequency (D), contraction amplitude (E), and peak–peak interval variance (F) vs. increasing furosemide concentration plots with insets showing connected individual experiments (*N* = 6; data presented as mean ± SD; values for each concentration are compared against the paired control value; Student’s paired *t*-test). For all plots *P ≤ .05. (G)–(I) Contraction frequency (G), contraction amplitude (H), and peak–peak interval variance (I) vs. increasing furosemide concentration plots with insets showing connected individual experiments (*N* = 6; data presented as mean ± SD; values for each concentration are compared against the paired control value; Student’s paired *t*-test). For all plots *P ≤ .05 and ***P ≤ .001.

CaCCinh-A01 (10^−5^ M), a more selective inhibitor of CaCCs, reduced the frequency and amplitude of contractions significantly (Figure 8A, C, and D). Peak–peak interval variance and RP diameter increased in response to 10^−6^–10^−5^ M CaCCInh-A01, suggesting that higher concentrations result in a loss of rhythmic contractions and loss of tone, respectively (Figure 8E and F). SMC-GCaMP3 preparations exposed to 5 µM CaCCInh-A01 caused rapid reductions in proximal RP Ca^2+^ wave frequency and near loss of resolvable Ca^2+^ waves in the distal pelvis (Figure 8B). In the presence of 5 µM CaCCInh-A01, Ca^2+^ wave propagation was decreased significantly vs. control (Figure 8G; *P*< .0001, control: 79.84 ± 24.72% vs. 5 µM CaCCInh-A01: 33.33 ± 19.33%, number of control values = 280, number of CaCCInh-A01 values = 155, *N* = 7 for each group). A spatio-temporal map from one experiment shows a reduction in the number of Ca^2+^ waves propagating the entire length of the RP (Figure 8H). Ca^2+^ wave frequency decreased significantly in the proximal (*P* < .001, *N* = 7 for each group) and distal (*P* < .0001) RP in the presence of CaCCinh-A01 (Figure 8I). Traces in Figure 8J show a large reduction in the frequency of Ca^2+^ waves in the proximal and distal pelvis. CaCCInh-A01 inhibits CaCC, but previous reports have also suggested concentration-dependent effects on L-type Ca^2+^ channels^34^. Therefore, we tested the effects of 10^−6^ and 10^−5^ M CaCCInh-A01 on K^+^ induced contractions (60 mM KCl). CaCCInh-A01 (10^−6^ or 10^−5^ M) did not significantly reduce responses to 60 mM KCl solution (Figure 8K; control: 28% ± 5% decrease, 10^−6^ M: 25 ± 4% decrease, 10^−5^ M: 25 ± 3% decrease, *N* = 5 for each group, *P* = .25 for control vs. 10^−6^ M paired *t*-test, *P* = .31 for control vs. 10^−5^ M). To further support a role for CaCCs in RP contraction propagation, we used two different Na^+^/K^+^/2Cl^−^ cotransporter (NKCC) inhibitors, furosemide and bumetanide. NKCC is important for intracellular Cl^−^ accumulation and, therefore, determines the outward Cl^−^ gradient^35^. Although we tested both compounds, it is well-documented that bumetanide is more potent vs. furosemide^36^. In gastrointestinal interstitial cells of Cajal, use of bumetanide blocks CaCC spontaneous inward currents^37^. Since furosemide and bumetanide are both solubilized in ethanol, we first performed vehicle control experiments to determine effects, if any, of ethanol alone (Figure 9A–C). Independent of concentration, ethanol did not significantly change the contraction frequency, amplitude or peak–peak interval variance (Figure 9A–C). However, furosemide significantly decreased contraction frequency at 10^−5^ M (Figure 9D), but did not affect the contraction amplitude (Figure 9E) or peak–peak interval variance (Figure 9F). More significant effects were observed with bumetanide (Figure 9G–I). At 10^−5^ M, bumetanide significantly decreased contraction frequency (Figure 9G) and significantly increased peak–peak interval variance (Figure 9I), demonstrating that contractions become irregular.

**Figure 10.**
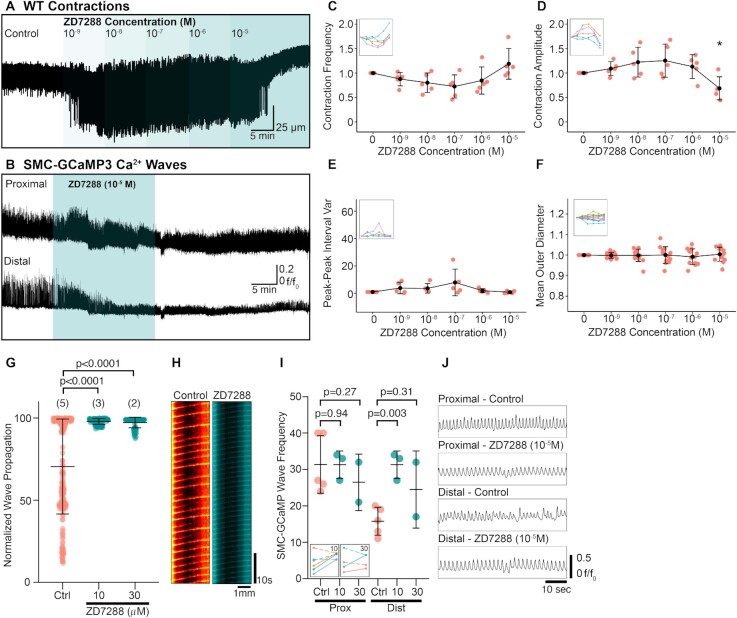
The pan-HCN channel antagonist, ZD7288 has limited effects on RP contractile activity but enhances peristaltic proximal to distal propagation. (A) Exemplar WT contraction trace demonstrating the effect of increasing concentrations of ZD7288 (10^−9^–10^−5^ M). (B) Ca^2+^ wave traces derived from the proximal and distal RP transiently treated with ZD7288 (10^−5^ M). (C)–(F) Contraction frequency (C), contraction amplitude (D), peak–peak interval variance (E), and mean outer RP diameter (F) vs. increasing ZD7288 concentration plots with insets showing connected individual experiments (*N* = 5 for panels (C)–(E), *N* = 10 for panel (F); data presented as mean ± SD; values for each concentration are compared against the paired control value; Student’s paired *t*-test). For all plots *P ≤ .05. (G) Normalized wave propagation plot for control and ZD7288 (10 and 30 µM)-treated SMC-GCaMP3 preparations. Each point represents one Ca^2+^ wave measurement. *N* = 5 for control group, *N* = 3 for 10 µM ZD7288 group, and *N* = 2 for 30 µM ZD7288 group. (H) Exemplar spatio-temporal maps showing Ca^2+^ waves propagating the length of RP in preparations incubated in control and ZD7288 (10 µM) solutions. (I) SMC-GCaMP3 Ca^2+^ wave frequency plot measured from proximal (Prox) and distal (Dist) regions in the presence of control (Ctrl), 10 µM ZD7288 (10), and 30 µM ZD7288 (3). Two inset show individual experiments from proximal (dotted lines) and distal (solid lines) regions in the presence of 10 and 30 µM ZD7288. (J) Representative line traces of Ca^2+^ waves sampled from proximal or distal regions treated with control or ZD7288 (10^−5^ M) solutions. Traces between conditions are sampled from the same region on the RP.

A previous study reported that ZD7288 (30 µM), a pan-HCN antagonist, disrupts contractile regularity of the RP and eventually abolishes contractions^26^. Therefore, we tested possible contributions from HCN on propagating peristaltic contractions. The frequency of RP contractions decreased transiently in response to ZD7288, but the amplitude of contractions increased at doses of 10^−8^–10^−6^ M (Figure 10A, C, and D). Contraction frequency increased and amplitude decreased significantly at 10^−5^ M ZD7288 (Figure 10A, C, and D). There was a tendency for the peak–peak interval variance to increase during 10^−7^ M, suggesting peristaltic contractions became irregular, but at higher concentrations (10^−6^–10^−5^ M) contractions were rhythmic (Figure 10F). There was no significant change in the diameter of the RP (Figure 10E), suggesting that increasing ZD7288 concentration does not reduce tone. In SMC-GCaMP3 preparations, ZD7288 (10^−5^ M) reduced the amplitude of Ca^2+^ waves in the distal RP and increased their frequency but had no effect on proximal Ca^2+^ wave frequency or amplitude (Figure 10B). A total of two concentrations of ZD7288 (10 and 30 µM) were tested on the length and frequency of propagating Ca^2+^ waves. Both concentrations potentiated the propagation of Ca^2+^ waves (Figure 10G), such that nearly all waves propagated along the entire length of the RP (Figure 10G; *P*< .0001, control: 70.48 ± 28.84% vs. 10 µM ZD7288: 97.91 ± 1.76%, *P*< .0001 control vs. 30 µM ZD7288: 97.20 ± 3.20%, number of control values = 262, number of 10 µM ZD7288 values = 121, number of 30 µM ZD7288 = 85, *N* = 5 for control group, *N* = 3 for 10 µM ZD7288 group, and *N* = 2 for 30 µM ZD7288). A spatio-temporal map from one experiment illustrates the increase in propagation length of Ca^2+^ waves in the presence of ZD7288 (10 µM; Figure 10H). ZD7288 (10 or 30 µM) did not affect proximal pelvis Ca^2+^ wave frequency (Figure 10I; control vs. 10 µM ZD7288: *P* = .94, *N* = 3, control vs. 30 µM ZD7288: *P* = .27, *N* = 2). However, the frequency of distal Ca^2+^ waves was increased significantly in the presence of ZD7288 (10 µM; Figure 10I; control vs. 10 µM ZD7288: *P* = .003, control vs. 30 µM ZD7288: *P* = .31), reflecting the increase in the length of Ca^2+^ wave propagation in the presence of ZD7288. Panels in Figure 10J show that proximal Ca^2+^ waves do not change, whereas distal waves become more regular and frequent.

## Discussion

In this study, we utilized wild-type and transgenic SMC-GCaMP3 mice to elucidate the propagation mechanisms of pyeloureteric peristaltic contractions. We visualized contractile responses to increasing concentrations of several ion channel antagonists. To complement this approach and to understand mechanisms that underlie peristaltic wave generation and propagation, we monitored the movement of Ca^2+^ waves along the length of the RP. We found that inhibition of voltage-gated Ca^2+^ channels had variable effects on Ca^2+^ wave frequency and propagation distance. Contractions and Ca^2+^ waves proved resistant to nicardipine, however, another L-type Ca^2+^ antagonist, isradipine, abolished propagating Ca^2+^ waves and contractions. Inhibiting T-type Ca^2+^ channels also reduced the frequency and propagation distance of Ca^2+^ waves. Notably, antagonists of ANO1 channels also decreased the propagation distance of Ca^2+^ waves and, in some cases, inhibited peristaltic propagation to the distal pelvis. Changing the chloride gradient pharmacologically with bumetanide decreased contraction frequency and caused irregular contractions. Rather than blocking generation and/or propagation of Ca^2+^ waves as previously reported^26^, an HCN channel antagonist increased the number of events propagating from proximal to distal pelvis (i.e., appeared to increase the safety factor for propagation).

The mechanisms that initiate activation of pacemakers to drive smooth muscle cell peristalsis in the RP are not fully understood, but CaCCs may provide a means to initiate contractions. In some smooth muscle tissues, transient increases in intracellular Ca^2+^ serve as the fundamental pacemaker signal in specialized interstitial cells, typically via activation of Ca^2+^-dependent ionic conductances^38–44^. PIC1s (classically referred to as atypical smooth muscle cells^1,10^), pacemakers of the RP, are abundant in the PKJ where contractions initiate. PIC1s express ANO1 and exhibit spontaneous endoplasmic reticulum-mediated Ca^2+^ transients^18^. The Ca^2+^ transients likely couple to openings of ANO1 channels to produce spontaneous transient inward currents (STICs). STICs have been reported in isolated atypical smooth muscle cells and are thought to coalesce into large inward currents to trigger spontaneous transient depolarizations^6^. Spontaneous transient depolarizations are likely the depolarization event that activate voltage-gated Ca^2+^ channels, facilitating the propagation of peristaltic contractions from the PKJ to the ureter. Previous pharmacological assays investigating the role of CaCCs in the RP led to inconsistent results, possibly due to the use of nonspecific Cl^−^ channel antagonists. For example, 4,4′‐diisothiocyano‐2,2′‐stilbenedisulfonic acid did not affect spontaneous transient depolarization frequency in murine proximal RP^7^. However, niflumic acid (NFA) reduced the frequency of events in guinea pig proximal RP^45^ and reduced STICs in atypical smooth muscle cells ^6^. Second generation ANO1 inhibitors, such as benzbromarone and CaCCinh-A01, have been used to evaluate the role of CaCCs in pacemaker activity in the gastrointestinal tract^46–48^, lymphatics^42^, and urethra^49,50^. In the present study, we found that these antagonists had significant effects on the propagating Ca^2+^ waves that underlie RP contractions. Although we found that CaCCInh-A01 inhibition had no significant effects on KCl-induced contractions, this does not completely exclude possible inhibition of L-type Ca^2+^ channels. Even a small inhibition of L-type Ca^2+^ channels by CaCCInh-A01 could be masked in largely depolarized cells. Further studies should consider using isolated RP smooth muscle cells to determine effect on L-type Ca^2+^ channel conductance. Despite this caveat, in combination with furosemide and bumetanide experiments, these data suggest that CaCCs reinforce the propagation of peristaltic contractions, thereby increasing the safety factor for propagation from the proximal to distal regions. Since ANO1 channels are expressed in PICs, we speculate that these cells provide a means of boosting inward currents and facilitate active peristaltic propagation^18^.

The role of voltage-gated Ca^2+^ channels in regulating RP peristalsis is also emerging. In other visceral organs and the sinoatrial node, L-type Ca^2+^ channels (i.e., Ca_V_1.2 and Ca_V_1.3) and low-voltage-gated T-type Ca^2+^ channels are involved in pacemaker activity^51–55^,^56–59^. In the RP, L-type Ca^2+^ channel expression has not been demonstrated immunohistochemically due to the lack of specific antibodies for channel subtypes, however, T-type Ca^2+^ channels expression has been determined in multiple species. T-type Ca^2+^ channels are present in murine PKJ^27,60^ and porcine and human calyces^25^. Previous studies suggest T-type expression is location dependent with higher expression of Ca_V_3.1 prevailing in the PKJ and tapering off in the mid-distal RP^27^. Similar expression patterns also exist for the other Ca_V_3 subtypes. Based on molecular expression data, Ca_V_3.2 and Ca_V_3.3 are more highly expressed in the distal vs. the proximal pelvis^18^. Collectively this suggests that T-type Ca^2+^ channels may be important in typical smooth muscle cells rather than PIC1s, although more cell-specific data is required to support this conclusion. The functional significance of T-type expression has also been previously investigated. T-type antagonists are known to reduce the frequency of pelvic contractions^27,60^. Although we also observe that T-type Ca^2+^ channels inhibition reduces contraction frequency, our SMC-GCaMP3 imaging data demonstrates that T-type Ca^2+^ channels are also required for ensuring proximal-to-distal peristaltic propagation (i.e., contribute to the safety factor for propagation). In our study, we found that TTA-A2, a potent, voltage-dependent, pan- T-type Ca^2+^ channels antagonist^32^ significantly reduced peristaltic transmission to the distal pelvis. We postulate that T-type Ca^2+^ channels expression in typical smooth muscle cells may promote propagation of Ca^2+^ waves associated with peristalsis. This is particularly important in the distal RP that has more negative resting potentials compared to the proximal region^13^. Together, T-type Ca^2+^ and CaCC channels may contribute toward providing more depolarized membrane potentials in the distal RP. This mechanism may be important once a propagating action potential reaches the distal portions of the RP. Due to increasing expression of 4-AP sensitive K^+^ channels in the distal RP, membrane potential is more hyperpolarized^13,14^. Therefore, we speculate that T-type Ca^2+^ channels and CaCCs will dictate if a wavefront passes through to the ureter.

Although T-type Ca^2+^ channels inhibition had robust effects on contractions, similar to other reports, we found that dihydropyridines which antagonize LTCCs had variable effects on RP contractions^7,8,60^. One study demonstrated that nifedipine decreased RP contraction frequency, action potential discharge, and caused membrane depolarization^7^. However, in another study by the same group, nifedipine either totally abolished or had minimal effects on Ca^2+^ waves in Ca^2+^-dye-loaded pelvis preparations^7^. In our study, we found that high concentrations (up to 10^−4^ M) of nicardipine failed to elicit significant effects on contractile properties. During our SMC-GCaMP3 assays, we also found that nicardipine (10^−6^ M) did not reduce Ca^2+^ wave propagation or affected proximal or distal Ca^2+^ wave frequency. The negligible effects of nicardipine contrasted sharply with the rapid inhibitory effects of isradipine on Ca^2+^ waves. One possible explanation may be T-type Ca^2+^ channel affinity for nicardipine and isradipine. A study by Perez-Reyes and colleagues found that isradipine potently inhibits T-type Ca^2+^ channels with an IC50 of < 3 μM^61^. Our data suggests that concentrations between 1 and 10 μM are sufficient to significantly inhibit and abolish contractions. Another explanation for difference between nicardipine and isradipine effects may be attributed to the specific repertoire of Ca_V_1 family channels expressed in the RP. Previous molecular characterization of the RP revealed that transcript levels of *Cacna1d* (encoding the α subunit of Ca_V_1.3) are more abundant in the proximal and distal pelvis, as compared to expression of *Cacna1c*^18^ (encoding the α subunit of Ca_V_1.2). The sensitivity of Ca_V_1 subtypes to dihydropyridines in RP smooth muscle cells has not been extensively characterized, but Ca_V_1.3 channels may contribute more substantially to excitation–contraction coupling in the pelvis than Ca_V_1.2 channels. Ca_V_1.3 channels activate at more negative potentials as compared to Ca_V_1.2 channels^62^, so Ca_V_1.3 may provide an additional safety factor to ensure that contractions initiated in the proximal RP propagate through to the ureter. This mechanism may initiate excitation-contraction coupling at lower levels of depolarization, ensuring that the excitatory events in proximal pacemaker cells propagate through to the distal pelvis. Future studies should attempt to determine whether Ca_V_1.2 and/or Ca_V_1.3 are important for excitation–contraction coupling in the RP. The contributions of Ca_V_1.2 and Ca_V_1.3 will need to be elucidated in studies where the dihydropyridine sensitivity of these channels has been genetically modified^63^.

The pacemaker channel HCN3, has been proposed as a conductance responsible for coordinating and triggering pyeloureteric peristalsis^25–27^. The pan HCN inhibitor ZD7288 abolishes unidirectional contractions and causes a loss of electrical activity in the PKJ, where HCN3 is expressed, in both unicalyceal (e.g., mice)^26^ and multicalyceal species (e.g., humans and pigs)^25^. ZD7288 is effective as an antagonist for HCN channels (IC_50_: 15 µM)^64^, but nonspecific effects on I_Ca_ and I_Na_ have also been reported^64^. For example, low concentrations of ZD7288 (< 1 µM) reduced I_Ca_ current sinoatrial nodal cells^65^, and higher concentrations (1–30 µM) reduced I_Na_ current in dorsal root ganglion neurons^64^. In our study, ZD7288 did not significantly affect contraction frequency or amplitude at low concentrations, and higher concentrations of ZD7288 (10 or 30 µM) potentiated the propagation of Ca^2+^ waves in the distal RP, such that the majority of Ca^2+^ waves propagated from the PKJ to the ureter. Thus, our observations differ significantly from the effects attributed to this compound in prior reports. Contrastingly, we did not observe uncoordinated peristalsis (i.e., retrograde peristalsis, ectopic initiation sites) at any ZD7288 concentration tested. A possible explanation for the differences in our observations and previous observations could be that we recorded propagating Ca^2+^ waves associated with peristalsis, whereas previous reports utilized voltage-sensitive dyes to monitor depolarizations in the RP. The voltage-sensitive dyes used in prior studies suffer from low sensitivity and resolution and could possibly have resulted in misinterpretations of the effects of the drugs. Voltage-sensitive dyes are best used for measuring large changes in membrane potential, which may not be a feature of many smooth muscle tissues. If ZD7288 affects the magnitude of I_Ca_, which is likely to be a feature of the electrical activity of cells in the RP, then it may have been difficult to detect these events in the presence of this drug. We also speculate that ZD7288 may block K^+^ conductances since we observe an improvement in the number of Ca^2+^ waves associated with peristalsis traveling down to the ureter. Additional experiments using conditional knock down of HCN3 will be necessary to clarify the role of this channel in peristaltic contraction modulation.

This study demonstrates improved approaches for investigating the mechanisms of propagation of peristaltic contractions in the RP using two imaging modalities and analyses. These tools will be valuable as means of evaluating other pharmacological compounds, genetic interventions, and the consequences of pathophysiological challenges. Our preparations maintain coupling between the proximal and distal RP, a feature typically missing in muscle strip studies. However, whilst other studies have carried out experiments with the RP intact^26,27,31,66^, our SMC-GCaMP3 assay provides greater spatial and temporal resolution to track propagating Ca^2+^ waves associated with contraction from the proximal RP through to the ureter. Our experiments have revealed novel and variable effects of L-type Ca^2+^ channel antagonists, nicardipine and isradipine, that seem likely due to different contributions of voltage-gated Ca^2+^ channels and effects of isradipine on T-type Ca^2+^ channels. We also revealed that T-type Ca^2+^ channels and CaCCs contribute to the safety factor for propagation of peristaltic contractions from the proximal pacemakers to the ureter. The integrated output of several inward current conductances is likely to be important for efficient transport of urine in the RP. Boluses of urine are carried to the ureter with each peristaltic wave. If the probability of bolus transport is reduced by incomplete peristalsis, this could negatively impact hydrostatic pressure in the renal papilla and the tubulointerstitium upstream, causing an overall loss of nephrons. Future studies using cell-specific genetic ablation of T-type Ca^2+^ channels and CaCCs will be important to acknowledge which cells and conductances provide the physiological safety factor for peristaltic contractions in the RP.

## Supplementary Material

zqac041_Supplemental_Video

## Data Availability

The data underlying this article will be shared on reasonable request to the corresponding author.
